# Effect of novel botanical synergist on the effectiveness and residue behavior of prothioconazole in wheat field

**DOI:** 10.1038/s41598-023-47797-z

**Published:** 2023-11-21

**Authors:** Yalin Wu, Yuanjian Yin, Xin Chen, Yeping Zhou, Shan Jiang, Mingming Zhang, Guangcheng Cai, Quan Gao

**Affiliations:** 1 Anhui Province Key Laboratory of Crop Integrated Pest Management, School of Plant Protection, Anhui Agricultural University, Hefei, China; 2 Anhui Province Engineering Laboratory for Green Pesticide Development and Application, School of Plant Protection, Anhui Agricultural University, Hefei, China; 3 Comprehensive Agricultural Service Station of Huoqiu County, Luan, China; 4 Fengtai Station of Plant Protection and Quarantine, Huainan, China

**Keywords:** Agroecology, Mass spectrometry

## Abstract

*Fusarium* head blight (FHB) is a critical fungal disease causes serious grain yield losses and mycotoxin contaminations. Currently, utilization of chemical fungicides is the main control method which has led to serious resistance. Development of novel synergist is an important strategy to reduce the usage of chemical fungicides and postpone the development of resistance, while natural components are interesting resources. In this study, the synergistic effect of *Taxodium 'zhongshansha'* essential oil (TZEO) was determined and the best synergistic ratio (SR) of 3.96 in laboratory which was observed when the weight ratio of TZEO and prothioconazole was 1 : 1 with the corresponding EC_50_ (half maximal effective concentration) value of *Fusarium graminearum* was 0.280 mg L^−1^. Subsequently, an increase of 6.31% on the control effect to FHB index in field test was observed when compared to the treatment with prothioconazole alone, though there was no significant difference between these treatments. Furthermore, we established an effective method to detect the mycotoxin contaminations in wheat grain with the limits of quantifications (LOQs) value of 5 µg kg^−1^ (DON, ZEN, 3-DON, and 15-DON) and 1 µg kg^−1^ (OTA) and the contents were less to the maximum residue limit (MRL) values. It was also shown that the application of 20% TZEO EW led to a 20% reduction in the use of prothioconazole, which was calculated based on the control effect values of 86.41% and 90.20% between the treatments of 30% prothioconazole OD (225 g a.i ha^−1^, recommend dosage) and 30% prothioconazole OD (180 g a.i ha^−1^) + 20% TZEO EW (225 mL ha^−1^), significantly. The initial residue of prothioconazole and prothioconazole-desthio was increased in the treatment with TZEO, which may play an important role in the synergistic effect on FHB. Moreover, none of the treatments posed a prothioconazole residue risk in the wheat grain and the environment. In addition, the essential oil has no any negative influence on wheat growth, which was revealed by a study of the chlorophyll content. These results provide an important botanical synergist for use with prothioconazole to control *Fusarium* head blight, and in-depth study to the synergistic mechanism of this oil is necessary in our future research.

## Introduction

Wheat (*Triticum aestivum* L.), one of the most important cereal crops in the world, accounts for approximately 30% of average global grain consumption^1^. *Fusarium* head blight (FHB) is a common fungal disease of wheat caused by the *Fusarium graminearum*, which has a serious impact on grain yield and quality^2^. FHB also can lead to a serious contamination of the affected grains with mycotoxins such as deoxynivalenol (DON) and zearalenone (ZEN), which are toxic to humans and animals^3,4^. Chemical control to this fungal disease is an important and frequently-used method, which will lead to some serious risks including resistance and residue^5,6^.

It can be concluded that the development of resistance is the most important challenge to control FHB, the proportion of carbendazim-resistant strains in Anhui and Jiangsu provinces was reported to be 12.8% and 43.3%, which is caused by the long-term use of the fungicide in these provinces^7^. Moreover, carbendazim stress leads to the production of more mycotoxins^8^. At present, there are more than 300 kinds of fungicides have been registered for the control of FHB^9^. The triazolinthione fungicide prothioconazole is a broad-spectrum systemic demethylase inhibitor, which can be used in the control of several diseases of cereal grain crops^10^. However, it has been determined that prothioconazole and the main metabolite (prothioconazole-desthio) exhibit potent teratogenicity and endocrine-disrupting effects on mammals^11^. It is important to establish an effective strategy to reduce the application of prothioconazole and improve its effectiveness, which may be attained by the development and use of novel botanical synergists.

In China, some synergists have been in use for a long time, castor oil is widely used due to the excellent solubility^12^. Karanja oil also exhibits significant synergistic effects to specific organochlorine fungicides in the control of stored grain pests^13^. Moreover, *Melaleuca alternifolia* oil, eucalyptus oil, *Cymbopogon citratus* oil and some phytochemicals including green tea polyphenols, eugenol, or geraniol can be used as synergists with several chemical pesticides^14,15^. In our previous study, we have determined the insecticidal activities of *Taxodium 'zhongshansha'* essential oil (TZEO) which was extracted from *T. 'zhongshansha'* leaves by hydrodistillation method, while study to the synergistic effect of this oil is limited^16^.

This study aims to determine the synergistic effect of TZEO on *F. graminearum* in our laboratory. Then, it is followed by a field test to evaluate the effectiveness of a mixture of 20% TZEO EW (emulsion in water) and 30% prothioconazole OD (oil dispersion) in controlling FHB. Furthermore, the effect of this oil on wheat growth was assessed along with mycotoxin contamination. Finally, the residue situations of prothioconazole and prothioconazole-desthio in wheat were determined. These results provide an insight into the use of *T. 'zhongshansha'* essential oil as a green fungicide synergist, which will provide a creative strategy to reduce the application of chemical fungicides used to control FHB and postpone the development of resistance. In addition, further studies are needed to elucidate the synergistic mechanism responsible for its activity.

## Results

### Synergistic effect of *T. ‘zhongshansha’* essential oil to prothioconazole in vitro

There was no obvious inhibitory effect of this oil against *F. graminearum* can be found when we evaluate the antifungal activity of TZEO (Table S1). A positive dosage effect was observed between the prothioconazole concentration and inhibitory rate, with an EC_50_ value of 1.124 mg L^−1^ (Table S2).

**Figure 1:**
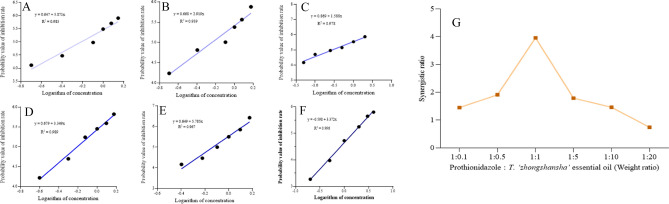
Optimal synergistic ratio of *Taxodium 'zhongshansha'* essential oil (TZEO) to prothionidazole against to *Fusarium graminearum*. (**A**–**F**) The linear regression between dosage and inhibitory rate when the ratio of prothioconazole : TZEO was 1 : 0.1, 0.5, 1, 5, 10, and 20, respectively, (**G**) the curve of synergistic ratios.

A preliminary evaluation of the synergistic effect of TZEO on prothioconazole was conducted with two dosages of the essential oil at 5 mg L^−1^ and 10 mg L^−1^. The EC_50_ of prothioconazole in the above treatments was determined as 0.607 and 0.940 mg L^−1^, respectively, which showed an obvious synergistic effect on prothioconazole for the inhibition of *F. graminearum* (Table S3). Based on the pre-experiment results, six treatments with different weight ratios of prothioconazole:TZEO (1:0.1, 1:0.5, 1:1, 1:5, 1:10, and 1:20) were performed. As shown in Fig. 1, the synergistic ratios were 1.45, 1.91, 3.96, 1.79, 1.46, and 0.74, with EC_50_ values of 0.780, 0.590, 0.280, 0.630, 0.770, and 1.500 mg L^−1^, respectively. The SR showed a trend of increasing first and then decreasing with weight ratio. In the all treatments, the best weight ratio between prothioconazole and TZEO was determined as 1:1, with a SR value of 3.96.

### Impact of *T. ‘Zhongshansha’* essential oil to the control of FHB in field study

**Figure 2:**
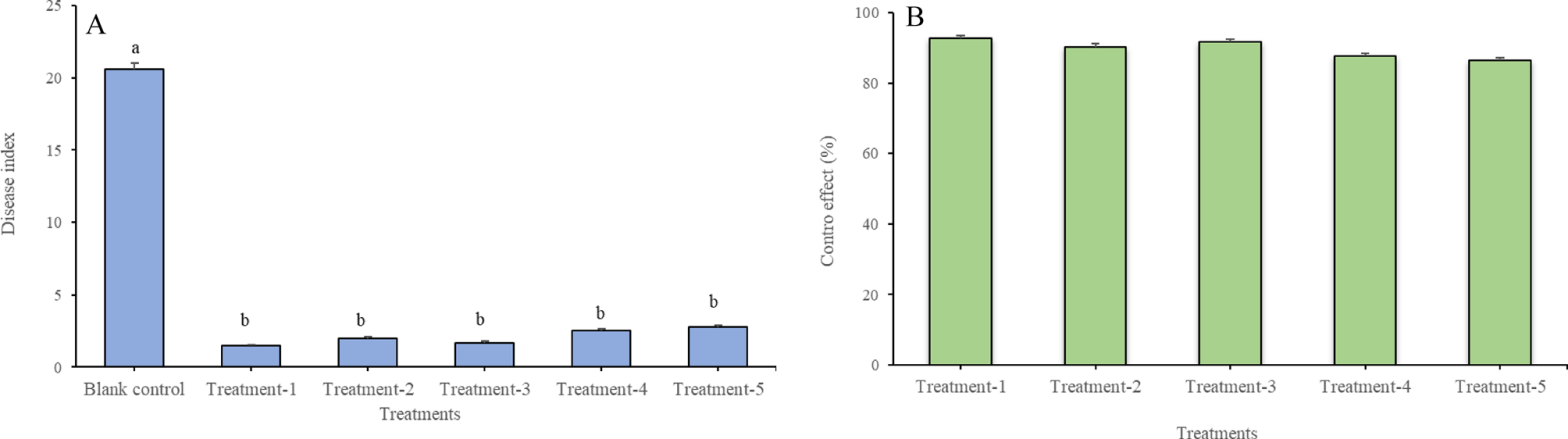
Control effect to *Fusarium* head blight in field test. (**A**) Disease index of *Fusarium* head blight in different treatments, (**B**) Control effect to *Fusarium* head blight in different treatments. Treatment-1, 30% prothioconazole OD (225 g a.i ha^−1^) + 20% TZEO EW (225 mL ha^−1^); Treatment-2, 30% prothioconazole OD (180 g a.i ha^−1^) + 20% TZEO EW (225 mL ha^−1^); Treatment-3, 30% prothioconazole OD (225 g a.i ha^−1^) + “Maifei” (225 mL ha^−1^); Treatment-4, 30% prothioconazole OD (180 g a.i ha^−1^) + “Maifei” (225 mL ha^−1^); Treatment-5, 30% prothioconazole OD (225 g a.i ha^−1^); Treatment-6, Blank control.

Based on the results in laboratory, a formulation of 20% TZEO EW was prepared for the field test (Fig. 2). Firstly, the use of TZEO has no negative influence on wheat growth, and none of the treatments were found to be associated with any phytotoxicity. This formulation showed an obvious synergistic effect on 30% prothioconazole OD according to the data obtained from the field tests which was repeated three times of each treatment. The disease index was 2.80 and the control effect value was 86.41% when only 30% prothioconazole OD (225 g a.i ha^−1^) was sprayed. In the treatments involving 30% prothioconazole OD (225 g a.i ha^−1^) + 20% TZEO EW (225 mL ha^−1^) and 30% prothioconazole OD (225 g a.i ha^−1^) + Maifei (225 mL ha^−1^), the control effect was 92.72% and 91.75%, with an increase of 6.31% and 3.20% compared to the treatment of 30% prothioconazole OD alone used, respectively. Moreover, the control effect in the treatment of 30% prothioconazole OD (180 g a.i ha^−1^) + 20% TZEO EW (225 mL ha^−1^) and 30% prothioconazole OD (180 g a.i ha^−1^) + Maifei (225 mL ha^−1^) was 90.20% and 87.62%, respectively, with corresponding disease index values of 2.00 and 2.55. Though there was no significant difference between these treatments, the results showed that TZEO was effective in increasing the control efficacy of prothioconazole on FHB. It can be concluded that the efficiency was better than that of the commercial synergist Maifei, which has been shown to reduce the application of prothioconazole by more than 20%.

In addition, we evaluated the influence on the chlorophyll content in leaves which can be used to growth condition of wheat using the SPAD value. The SPAD value of 30% prothioconazole OD (225 g a.i ha^−1^) + 20% TZEO EW (225 mL ha^−1^) and 30% prothioconazole OD (180 g a.i ha^−1^) + 20% TZEO EW (225 mL ha^−1^) was 35.42 and 39.72. In the two treatments with Maifei and different dosage of prothioconazole, the value was 37.38 and 40.84, respectively. When only prothioconazole was sprayed, the SPAD value was 38.71, while the value was 38.98 in the blank control. It can be concluded that there was no significant difference between all treatments (Fig. S1).

### Effect of *T. ‘zhongshansha’* essential oil on the control of mycotoxin contamination

In this study, the limit of quantification (LOQ) value of DON, ZEN, 3-DON, and 15-DON was 5 µg kg^−1^, while that for OTA was 1 µg kg^−1^, which was satisfied to the maximum residue limit (MRL) values of DON, ZEN, and OTA at 1000, 60, and 5 µg kg^−1^. The content of DON, 3A-DON, 15A-DON, and OTA in blank control was 1205.55 µg kg^−1^, 259.68 µg kg^−1^, 290.05 µg kg^−1^, and 640.96 µg kg^−1^, respectively (Table 1)^17^. When 30% prothioconazole OD (225 g a.i ha^−1^) + 20% TZEO EW (225 mL ha^−1^) was sprayed, only 41.03 µg kg^−1^ of 15A-DON was detected in the grains. DON (120.23 µg kg^−1^) and 15A-DON (50.15 µg kg^−1^) were found in the treatment of 30% prothioconazole OD (180 g a.i ha^−1^) + 20% TZEO EW (225 mL ha^−1^). Similarly, the content of 15A-DON was 52.35 µg kg^−1^ in the treatment of 30% prothioconazole OD (225 g a.i ha^−1^) + Maifei (225 mL ha^−1^), and the DON and 15A-DON content was 71.50 µg kg^−1^ and 51.60 µg kg^−1^ in the 30% prothioconazole OD (180 g a.i ha^−1^) + Maifei (225 mL ha^−1^). Moreover, DON content of 211.50 µg kg^−1^ and 15A-DON of 52.80 µg kg^−1^ were observed in the grains when only 30% prothioconazole OD (225 g a.i ha^−1^) was sprayed.

**Table 1:** Comparison of control effect to mycotoxin contamination in wheat grain.

Treatment	Application dosage	Mycotoxin (µg/kg)				
		DON	3A-DON	15A-DON	ZEN	OTA
30% prothioconazole OD + 20% TZEO EW	225 g a.i ha^−1^ + 225 mL ha^−1^	< LOQ	< LOQ	41.03	< LOQ	< LOQ
	180 g a.i ha^−1^ + 225 mL ha^−1^	120.33	< LOQ	50.15	< LOQ	< LOQ
30% prothioconazole OD + “Maifei”	225 g a.i ha^−1^ + 225 mL ha^−1^	< LOQ	< LOQ	52.35	< LOQ	< LOQ
	180 g a.i ha^−1^ + 225 mL ha^−1^	71.50	< LOQ	51.60	< LOQ	< LOQ
30% prothioconazole OD	225 g a.i ha^−1^	211.50	< LOQ	52.80	< LOQ	< LOQ
Blank control	–	1205.55	259.68	290.05	< LOQ	640.96

### Effect of *T. ‘zhongshansha’* essential oil on prothioconazole residues

**Figure 3:**
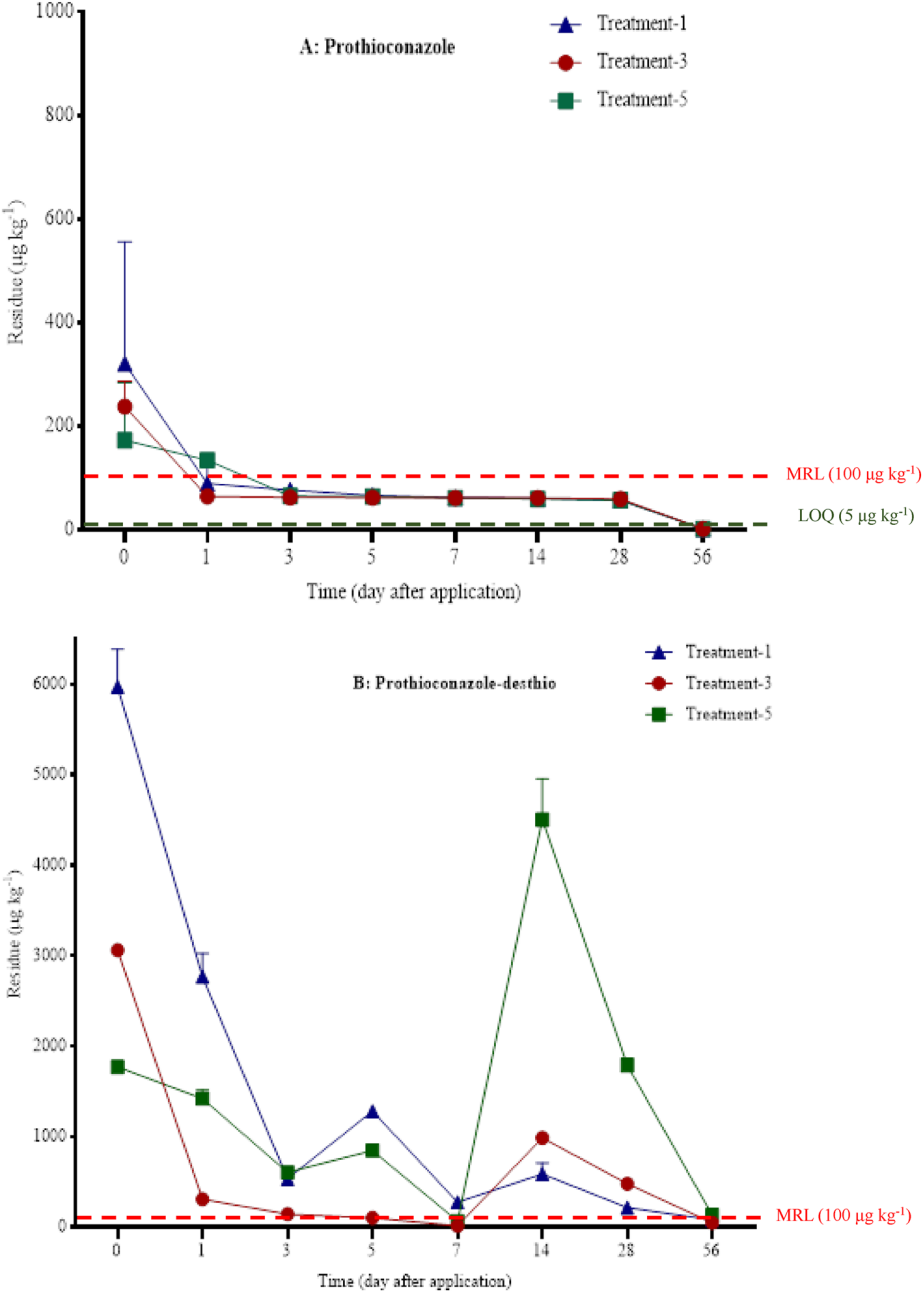
Residue dynamics of prothioconazole (**A**) and prothioconazole-desthio (**B**) in wheat leaf. Treatment-1, 30% prothioconazole OD (225 g a.i ha^−1^) + 20% TZEO EW (225 mL ha^−1^); Treatment-3, 30% prothioconazole OD (225 g a.i ha^−1^) + “Maifei” (225 mL ha^−1^); Treatment-5, 30% prothioconazole OD (225 g a.i ha^−1^).

As shown in Fig. 3A, the average initial residue of prothioconazole in wheat plants were 320.12 µg kg^−1^, 237.60 µg kg^−1^, and 172.12 µg kg^−1^ in the treatment with 30% prothioconazole OD (225 g a.i ha^−1^) + 20% TZEO EW (225 mL ha^−1^), 30% prothioconazole OD (225 g a.i ha^−1^) + Maifei (225 mL ha^−1^), and 30% prothioconazole OD (225 g a.i ha^−1^), respectively. The content of prothioconazole decreased quickly on day 2 after spraying 30% prothioconazole OD together with the synergist, a similar phenomenon was observed on day 4 when only 30% prothioconazole OD was applied. At the 28^th^ day, the average prothioconazole residue content after spayed 30% prothioconazole OD (225 g a.i ha^−1^) + Maifei (225 mL ha^−1^) was 58.84 µg kg^−1^, and the value was 56.70 µg kg^−1^ in the treatment of 30% prothioconazole OD (225 g a.i ha^−1^) + 20% TZEO EW (225 mL ha^−1^) and 30% prothioconazole OD. In addition, no prothioconazole was observed in all treatments on harvest day, while the value was less than LOQ (5 µg kg^−1^) in the soil and grain samples.

The initial concentration of prothioconazole-desthio in wheat plant was 5970.50 µg kg^−1^ in the treatment of 30% prothioconazole OD (225 g a.i ha^−1^) + 20% TZEO EW (225 mL ha^−1^), while the final residue value was 80.00 µg kg^−1^ (Fig. 3B). In the treatment of 30% prothioconazole OD (225 g a.i ha^−1^) + Maifei (225 mL ha^−1^) was sprayed, the initial residue of prothioconazole-desthio was 3062.54 µg kg^−1^ and the final value was 46.93 µg kg^−1^. The values of the initial and final residue concentration in the treatment of 30% prothioconazole OD were 1769.18 µg kg^−1^ and 129.80 µg kg^−1^, respectively. In addition, similar curves were observed in all treatments with two increasing trends on the 6th and 14th day, which may be because of the degradation and enrichment processes^18^. Moreover, the values of prothioconazole-desthio residue were less than LOQ in all solid samples, except the initial residue of 23.86 µg kg^−1^, 29.72 µg kg^−1^, and 26.72 µg kg^−1^ in treatment of 30% prothioconazole OD (225 g a.i ha^−1^) + 20% TZEO EW (225 mL ha^−1^), 30% prothioconazole OD (225 g a.i ha^−1^) + Maifei (225 mL ha^−1^), and 30% prothioconazole OD, respectively. Finally, it was determined that there was no prothioconazole-desthio residue in wheat grains.

## Discussion and conclusion

In this study, the inhibitory rate of *T. ‘zhongshansha’* essential oil against *F. graminearum* was 11.48% at a treatment concentration of 5 mg/L, and 13.26% with 10 mg/L. Hence, it is difficult to develop this oil as a novel botanical fungicide, while the development as synergist may be a creative strategy owing to the physicochemical properties of the essential oil^19,20^. Then, the best synergistic ratio of 3.96 was observed when the weight ratio of TZEO and prothioconazole was 1:1, with an EC_50_ of 0.280 mg L^−1^. It can be concluded that the synergistic effect of TZEO was significant, which was caused by the characteristic liposolubility of essential oil. It has been reported that various essential oils have obvious synergistic effects to some fungicides, including tea tree essential oil, *Cinnamomum cassia* oil, *Tagetes minut*a oil, and neem oil^21,22^. Only a limited number of studies have reported data regarding the actual effect in field studies, our results may provide a basis for the utilization of essential oils as novel botanical synergists.

Then, field test was used to verify the synergistic effect to prothioconazole in the control of *Fusarium* head blight and mycotoxin contaminations. Since mycotoxins in wheat grains have serious negative influences on human and animal health, one of the important evaluation indicators of fungicides used against FHB is the control effect to mycotoxins^23,24^. The occurrence of mycotoxins is unpredictable and difficult to estimate, which pose a serious health risk to animals and humans^25,26^. It was concluded that the use of the synergist in this study has a significant effect on the control of mycotoxin contamination, and prothioconazole was an effective agent to reduce mycotoxin content^27^.

The efficiency of the essential oil was better than the commercial synergist Maifei in field, which can result in a more than 20% reduction in the application of prothioconazole. It has been reported that the natural phenolic agent octyl gallate enhanced the activities of kresoxim-methyl and fludioxonil on *Penicillium expansum*^28^, and 2,3-dihydroxybenzaldehyde showed similar activity to amphotericin B^29^. Volatile components such as eugenol, thymol, and carvacrol synergistically interacted with fluconazole, which can increase the penetration of the fungicide by inhibiting biofilm formation and creating pores in the cell membrane^30^. Diferu-loylmethane enhanced the antifungal activities of azoles against *Candida albicans* by increasing the levels of reactive oxygen species and regulating the genes related to fungal oxidative stress, which may lead to apoptosis^31^. Moreover, the use of synergist was a creative strategy to postpone the development of resistance^32^. The treatment with DMIs (Demethylase inhibitors) increased the expression levels of erg11 (the target gene of DMI fungicides) by 15.4 to 56.6-fold, then the treatment with a mixture of schizostatin and DMIs reverted the erg11 transcription levels to the pre-DMI treatment levels^33,34^. In addition, this oil had no negative influence on the chlorophyll content. It has been reported that the spraying of some essential oils easily leads to dehydration, hence the result of this study showed that *T. ‘zhongshansha’* essential oil was safe to wheat growth^35,36^.

Finally, the TZEO also enhances the initial residue of prothioconazole and prothioconazole-desthio, which may play an important role in revealing the synergistic effect on FHB. It can be concluded that the use of synergist enhances the stability of this fungicide, which can be determined by the comparison of the initial residue content^37^. The use of a synergist has no residue risk, that was determined as the comparison with the MRL value of prothioconazole in wheat grain which was identified with the concentration of prothioconazole-desthio (100 μg kg^−1^) according to standard requirements^38,39^. As we all know, the differences between a larger scale in real agricultural model and our study are significant. Hence, more optimize study to this formulation and the related evaluation including in-depth exploration to the synergistic mechanism of this oil is necessary in our future research.

In summary, *T. ‘zhongshansha’* essential oil showed significant synergistic effect to prothioconazole against *F. graminearum* with the best synergistic ratio value of 3.96. This essential oil also showed obvious synergistic effect in the control of *Fusarium* head blight and mycotoxin contaminations, which was better than the commercial synergist Maifei in field, while also can result in a more than 20% reduction in the application of prothioconazole. In addition, the essential oil had no negative influence on the chlorophyll content, which demonstrate that *T. ‘zhongshansha’* essential oil was safe to wheat growth. This oil also enhanced the initial residue of prothioconazole and prothioconazole-desthio, which may play an important role in revealing the synergistic effect on FHB. These results provide a creative strategy for the use of *T. ‘zhongshansha’* essential oil as a green fungicide synergist, which is important to reduce the application of chemical fungicides and postpone the development of fungicide resistance. Future studies are needed for the elucidation of the synergistic mechanism and evaluation of the synergistic effect of this oil on other chemical fungicides.

## Materials and methods

### Chemicals and reagents

The standards of prothioconazole, prothioconazole-desthio (purity ≥ 97.00%), and 30% prothioconazole OD were purchased from Jiuyi Agriculture Co., Ltd (Anhui, China). Mycotoxin standards (purity ≥ 98.00%) including DON, 3-acetyldeoxynivalenol (3A-DON), 15A-DON (15-O-acetyl-4-deoxynivalenol), ZEN, and OTA were purchased from Sigma-Aldrich (Saint Louis, MO, USA).

TZEO was isolated from *T. ‘zhongshansha’* leaves via the hydrodistillation method with distilled water at a 1:8 (solid:liquid) ratio, 5 mL *n*-hexane was added into the flask with a 8 h extraction procedure. The extraction temperature was set at 100 °C, and volatile components were evaporated and dissolved in *n*‐hexane via inverse flow, continuously. Finally, the *n*-hexane layer was collected and concentrated in vacuo at room temperature which was transferred to a brown bottle for storage.

The 20% TZEO EW was obtained from Anhui Province Engineering Laboratory for Green Pesticide Development and Application, which was stored at a room temperature. HPLC-grade acetone and methanol were purchased from Aladdin (Shanghai, China), while acetonitrile was obtained from TEDIA (Cincinnati, OH, USA). All the analytic-grade solvents used in this study were purchased from Anpel (Shanghai, China). A commercial synergist named Maifei was provided by Guangyuan Yinong Chemical Co., Ltd (Beijing, China).

### Evaluation to synergistic effect of TZEO in laboratory

The phytopathogens *F. graminearum* was obtained from Anhui Province Engineering Laboratory for Green Pesticide Development and Application of Anhui Agricultural University. The phytopathogen was inoculated at the center of the plate and incubated in the dark at 28 °C. In addition, the strain was identified by Prof. Li Chen who has studied to the FHB decades. Firstly, we assayed the individual antifungal activity of prothioconazole and TZEO against *F. graminearum *in vitro via mycelial growth rate method. A standard solution of prothioconazole was diluted to prepare test solutions, the potato dextrose agar medium (PDA medium) was subsequently sub-packed in 6 sterilized petri dishes, with 100 mL of medium per dish. Based on the results of pre-experiments, the actual test dosage of prothioconazole was 8.0, 5.0, 2.5, 1.25, 0.625, 0.313, and 0.156 mg L^−1^. Simultaneously, the test dosage of TZEO was set as 20.0, 10.0, 5.0, 2.0, 1.0, and 0.5 mg L^−1^. The test solutions were prepared when we assayed the experiments, three replicates of every treatment were determined.

For evaluating the synergistic effect of TZEO and prothioconazole on the control of *F. graminearum* in laboratory, different concentrations of the essential oil were added into the fungicide solution with a volume ratio of 1:1, in order to obtain a combination solution. Based on the results of the pre-experiment, six treatments with a mass ratio of 1:0.1, 1:0.5, 1:1, 1:5, 1:10, and 1:20 (Prothioconazole : TZEO) were set up to evaluate the synergistic effect. Five dosages of every treatment were assayed and repeated 3 times. The treatment with prothioconazole alone was defined as control and the growth control (without any treatment) was set, simultaneously.

All treated plates were placed in the dark at 28 °C and photographed after 48 h. Thereafter, the synergistic effect was calculated by the synergistic ratio (SR). While SR value is more than 1 indicated synergism, a value near 1 indicated additive action and a value less than 1 indicated antagonism. The formula for the calculation of SR is as follows^40^:

$$ {\text{SR}} = \frac{{{\text{EC50}}\,\,\,{\text{of}}\,\,{\text{prothioconazole}}}}{{{\text{EC50}}\,\,{\text{of}}\,\,{\text{combination}}\,\,{\text{solution}}}} $$

### Field test design and sampling

The test was conducted in accordance with Standard Operating Procedures on Pesticide Registration Residue Field Trials^41^. Six treatments were used and the design was determined by the results in laboratory (Table S4). The agent and dosage were 30% prothioconazole OD (225 g a.i ha^−1^, recommend dosage), 30% prothioconazole OD (225 g a.i ha^−1^) + 20% TZEO EW (225 mL ha^−1^), 30% prothioconazole OD (180 g a.i/ha) + 20% TZEO EW (225 mL ha^−1^), 30% prothioconazole OD (225 g a.i ha^−1^) + Maifei (225 mL ha^−1^), 30% prothioconazole OD (180 g a.i ha^−1^) + Maifei (225 mL ha^−1^), and a blank control, respectively. All the test agents were sprayed using a UAV sprayer (P20, XAG Technology Co., Ltd., Guangzhou, China) with a spraying width of 5 m, the flying height and velocity was 2.6 m and 5 m s^−1^, respectively. Each plot measured 120 m (length) by 5 m (width) (total plot area = 600 m^2^) and separated by a buffer zone. The field test was assayed three replications of every treatment and conducted on flat terrain in Fengtai County, Anhui Province. The levels of soil fertility, cultivation and fertilization management were consistent with local agricultural production, which was satisfied to the requirements of this test. The first spraying was done at the beginning of the wheat flowering stage, and the second spraying was performed after 7 days.

For the residue risk assessment of prothioconazole under different treatment conditions, representative plant and soil samples in the treatment of 30% prothioconazole OD (225 g a.i ha^−1^, recommend dosage), 30% prothioconazole OD (225 g a.i ha^−1^) + 20% TZEO EW (225 mL ha^−1^), 30% prothioconazole OD (225 g a.i ha^−1^) + Maifei (225 mL ha^−1^) and blank control were collected at 2 h. Then, the samples were collected at 1st, 3rd, 5th, 7th, 10th, 14th, and 21st day after the application of fungicides. On the harvest day, the grain samples were collected along with the plant and soil samples for the final residue analysis. In all, samples of plant and soil weighing 5 kg were collected each time respectively and stored at − 20 °C until further use. The harvested grains (2 kg) from each treatment were obtained to determine the mycotoxin concentrations.

### Evaluation to the effectiveness in controlling to *Fusarium* head blight

Twenty days after application, we assessed the effect of these treatments on FHB disease symptom development using the disease scale. Five points were chosen according to the diagonal distribution in each plot, and one hundred wheat plants in each point were fixed to investigate the disease index of FHB. Firstly, FHB severity was determined based on the percentage of surfaces exhibiting visible symptoms, with the scores ranging from 0 to 7 (0, 0%; 1, 0–25%; 3, 25–50%; 5, 50–75%; 7, 75–100%)^42^. Subsequently, the disease index (DI) and control effect to the DI were obtained using the follow formulars^43^:

$$ {\text{Disease}}\,\,{\text{index}} = \frac{{\sum ({\text{Disease}}\,\,{\text{scale}}\,\, \times \,\,{\text{Total}}\,\,{\text{number}}\,{\text{of}}\,{\text{plants}}\,\,{\text{categorized}}\,{\text{in}}\,{\text{that}}\,{\text{scale}})}}{{{\text{The}}\,\,{\text{highest}}\,{\text{scale}}\, \times \,\,{\text{Total}}\,\,{\text{number}}\,\,{\text{of}}\,\,{\text{plants}}\,{\text{assessed}}\,\,{\text{per}}\,\,{\text{plot}}}} \times 100 $$

$$ {\text{Control}}\,\,{\text{effect}}\,\,(\% ) = \frac{{{\text{DI}}\,{\text{of}}\,\,{\text{blank}}\,\,{\text{control}}\,\,{\text{treatment}} - {\text{DI}}\,\,{\text{of}}\,{\text{Fungicide}}\,\,{\text{treatment}}}}{{{\text{DI}}\,{\text{of}}\,{\text{blank}}\,{\text{control}}\,{\text{treatment}}}} \times 100 $$

In addition, the chlorophyll content in wheat leaf was determined using a SPAD-502 Chlorophyll Meter Model (Konica Minolta, Chiyoda city, Tokyo metropolis, Japan) with SPAD value. Ten leaves of similar size and consistent growth were collected from each plot on the fifth day after application, five points of each leaf were assayed to calculate the average value. The plant collection and use was in accordance with all the relevant guidelines, including the national standard “Method for Determining Chlorophyll Content” GB/T 15401-1995.

### UPLC-MS/MS analysis of prothioconazole residue and mycotoxins

Based on the previous study^41^, the extraction and purification methods of prothioconazole and prothioconazole-desthio in different matrices were optimized. Two grams of the sample (plant, soil, and grain) was weighed and put into a centrifuge tube. After the addition of 5 mL deionized water and 10 mL acetonitrile, the sample was homogenized for 2 min. The extraction procedure was assayed with ultrasonic for 15 min. Four grams of NaCl was added and homogenized for 1 min. Centrifugation was subsequently carried out for 3 min at 20,627×*g*, following which 1 mL of supernatant was filtered through a 0.22-μm nylon membrane for UPLC–MS/MS analysis.

Liquid chromatography was carried out using a UPLC instrument (Waters, MA, USA) interfaced with a Xevo TQ-Smicro mass spectrometer (Waters, MA, USA). The mycotoxins and fungicides were separated using a UPLC® BEH C18 column (2.1 × 50 mm, 1.7 μm; Waters, MA, USA). Isocratic elution was carried out using water containing 0.1% (v/v) formic acid (A) and acetonitrile (B) at a flow rate of 0.4 mL min^−1^. The injection volume was 10 μL and column temperature was set at 40 °C. Mass spectrometric detection was performed in the multiple reaction monitoring (MRM) mode with electrospray negative ionization (ESI^−^, 3.0 kV). The main parameters were as follows (Fig. S2): cone source temperature, 150 °C; drying gas flow, 150 L h^−1^; sheath gas temperature, 500 °C; sheath gas flow, 1000 L h^−1^. For the detection of prothioconazole, the *m*/*z* value of the parent ion was 342.20, while the *m*/*z* values of quantification ions were 100.10 and 125.00. For prothioconazole-desthio, the parameters were as follows: parent ion, 312.20; quantification ions, 70.00 and 125.20.

To investigate mycotoxin contamination of grains, 10 g of grain powder was filtered through a 60-mesh sieve. It was transferred into a conical flask, followed by the addition of 20 mL water and 40 mL acetonitrile. Then, the solution was subsequently vibrated at 180 rpm for 10 min after homogenization for 1 min. 4 g of MgSO_4_ and 8 g of NaCl were added into a flask which was vibrated for 2 min. Two milliliter of the supernatant was transferred into a centrifuge tube. After centrifugation for 3 min at 20,627×*g*, the supernatant was filtered through a 0.22-μm nylon membrane for UPLC-MS/MS analysis. The *m*/*z* values of the observed UPLC-MS/MS peaks are detailed in Table S5, other detection conditions and parameters were the same as described above with electrospray positive ionization (ESI^+^, 3.0 kV) (Fig. S3).

### Statistical analysis

The data was expressed as mean ± standard deviation (SD), and the differences in the mean values between treatment groups were calculated using SPSS 22.0 software (Chicago, IL, USA). DPS 7.5 software was used to calculate the EC_50_ value and its 95% confidence interval. Comparisons with *p* ≤ 0.05 were considered significantly different. All figures were created using GraphPad Prism 7 (San Diego, CA, USA).

### Supplementary Information

Supplementary Information.

## Data Availability

All data generated or analysed during this study are included in this published article and its supplementary information files.
