# Supplementary material for: Effect of novel botanical synergist on the effectiveness and residue behavior of prothioconazole in wheat field
Source: Sci Rep. 2023 Nov 21;13:20353. doi: 10.1038/s41598-023-47797-z (PMC10663447; doi:10.1038/s41598-023-47797-z)
Supplement: Supplementary file 1 — Supplementary Information. [file 41598_2023_47797_MOESM1_ESM.pdf]

Effect of novel botanical synergist on the effectiveness and residue behavior of prothioconazole in wheat field

Yalin Wu[1,2,†], Yuanjian Yin[3,†], Xin Chen[1,2], Yeping Zhou[1,2], Shan Jiang[4], Mingming Zhang[4], Guangcheng Cai[4] & Quan Gao[1,2,*]

1 Anhui Province Key Laboratory of Crop Integrated Pest Management, School of Plant Protection, Anhui Agricultural University, Hefei, China

2 Anhui Province Engineering Laboratory for Green Pesticide Development and Application, School of Plant Protection, Anhui Agricultural University, Hefei, China

3 Comprehensive Agricultural Service Station of Huoqiu County, Luan, China

4 Fengtai Station of Plant Protection and Quarantine, Huainan, China

* quangao@ahau.edu.cn

Supplementary information

Table S1. Antifungal activity of *T. 'zhongshansha'* essential oil against *F. graminearum* (48 h).

Table S2. The antifungal activity of prothioconazole against *F. graminearum* (48 h).

Table S3. The pre-experiment results of evaluation to synergistic effect of *T. 'zhongshansha'* essential oil to prothioconazole against *F. graminearum* (48 h).

Table S4. Design and layout of experimental treatments.

Table S5. Tandem mass spectrometry parameters of the mycotoxins.

Figure S1. Treatment-1, 30% prothioconazole OD (225 g a.i ha^{-1}) + 20% TZEO EW (225 mL ha^{-1}); Treatment-2, 30% prothioconazole OD (180 g a.i ha^{-1}) + 20% TZEO EW (225 mL ha^{-1}); Treatment-3, 30% prothioconazole OD (225 g a.i ha^{-1}) + "Maifei" (225 mL ha^{-1}); Treatment-4, 30% prothioconazole OD (180 g a.i ha^{-1}) + "Maifei" (225 mL ha^{-1}); Treatment-5, 30% prothioconazole OD (225 g a.i ha^{-1}); Treatment-6, Blank control.

Figure S2. Mass spectrogram about quantitative ion pair of prothioconazole and prothioconazole-desthio.

Figure S3. Mass spectrogram about quantitative ion pair of five mycotoxins.

Table S1. Antifungal activity of *T. 'zhongshansha'* essential oil against *F. graminearum* (48 h).

Concentration of *T. 'zhongshansha'* essential oil (mg L^{-1})	Inhibitory rate (%)	RSD (%)
0.5	3.48	0.47
1	7.64	1.42
2	8.86	2.04
5	11.4	1.48
10	13.26	1.68

Table S2. The antifungal activity of prothioconazole against *F. graminearum* (48 h).

Concentration of prothioconazole ($mg\ L^{-1}$)	Inhibitory rate (%)	RSD (%)
0.156	19.78	1.97
0.313	31.67	1.67
0.625	37.36	2.44
1.25	48.68	1.57
2.5	60.84	1.95
5	78.97	2.07
8	88.96	1.88

Table S3. The pre-experiment results of evaluation to synergistic effect of *T. 'zhongshansha'* essential oil to prothioconazole against *F. graminearum* (48 h).

Concentration of *T. 'zhongshansha'* essential oil (mg L^{-1})	Concentration of prothioconazole (mg/L)	Inhibitory rate (%)	EC_{50} (mg L^{-1})
5	0.156	24.29	0.607
	0.313	37.73	
	0.625	45.73	
	1.25	68.99	
	2.5	72.52	
	5	90.42	
10	0.156	24.06	0.940
	0.313	29.50	
	0.625	42.15	
	1.25	56.30	
	2.5	66.72	
	5	75.71	

Table S4. Design and layout of experimental treatments.

Treatment	Agent	Dosage
Treatment-1	30% prothioconazole OD + 20% TZEO EW	225 g a.i ha^{-1} +225 mL ha^{-1}
Treatment-2	30% prothioconazole OD + 20% TZEO EW	180 g a.i ha^{-1} +225 mL ha^{-1}
Treatment-3	30% prothioconazole OD + “Maifei” synergist	225 g a.i ha^{-1} +225 mL ha^{-1}
Treatment-4	30% prothioconazole OD + “Maifei” synergist	180 g a.i ha^{-1} +225 mL ha^{-1}
Treatment-5	30% prothioconazole OD	225 g a.i ha^{-1}
Treatment-6	Blank control	--

Table S5. Tandem mass spectrometry parameters of the mycotoxins.

Analyte	Precursor ion $[M+H]^+$	Product ion (*m/z*)	Fragment of voltage (V)	Collision energy (V)
DON	297.15	203.15/249.09*	20	10/16
3A-DON	319.12	203.10/231.10*	17	11/19
15A-DON	339.19	321.14/137.07*	17	13/13
ZEN	339.15	187.06/283.13*	18	9/7
OTA	404.12	358.12/239.04*	20	10/16

* the quantitative ion.

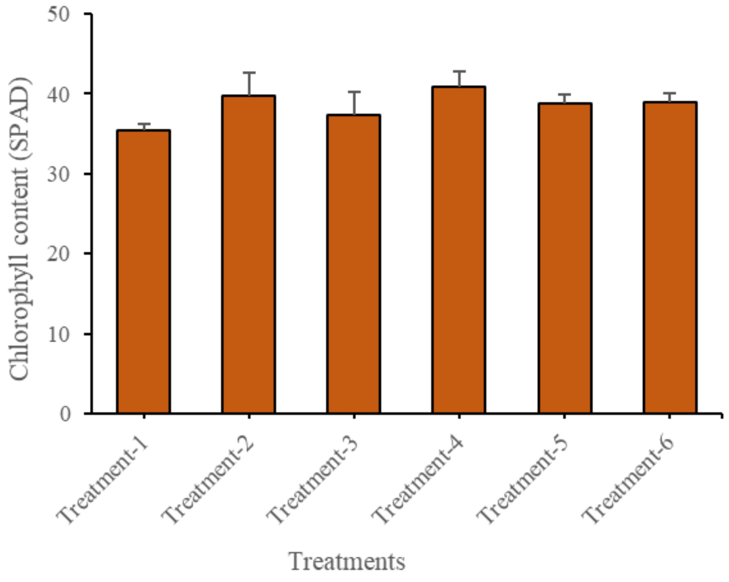

Figure S1. Treatment-1, 30% prothioconazole OD (225 g a.i ha^{-1}) + 20% TZEO EW (225 mL ha^{-1}); Treatment-2, 30% prothioconazole OD (180 g a.i ha^{-1}) + 20% TZEO EW (225 mL ha^{-1}); Treatment-3, 30% prothioconazole OD (225 g a.i ha^{-1}) + "Maifei" (225 mL ha^{-1}); Treatment-4, 30% prothioconazole OD (180 g a.i ha^{-1}) + "Maifei" (225 mL ha^{-1}); Treatment-5, 30% prothioconazole OD (225 g a.i ha^{-1}); Treatment-6, Blank control.

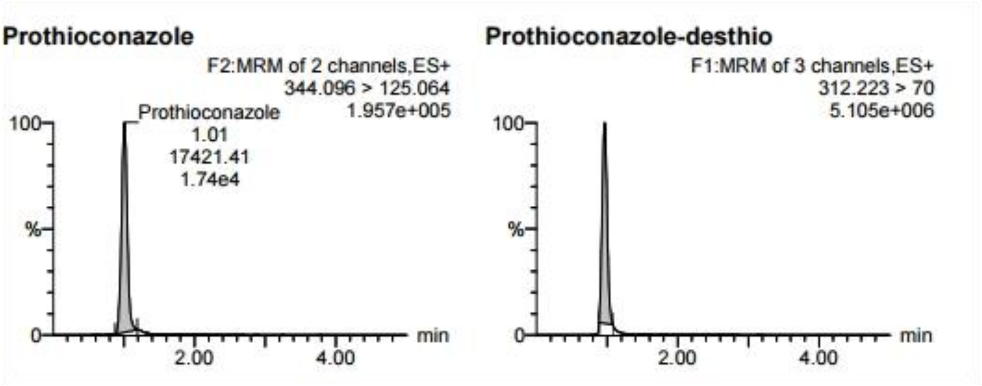

Figure S2. Mass spectrogram about quantitative ion pair of prothioconazole and prothioconazole-desthio.

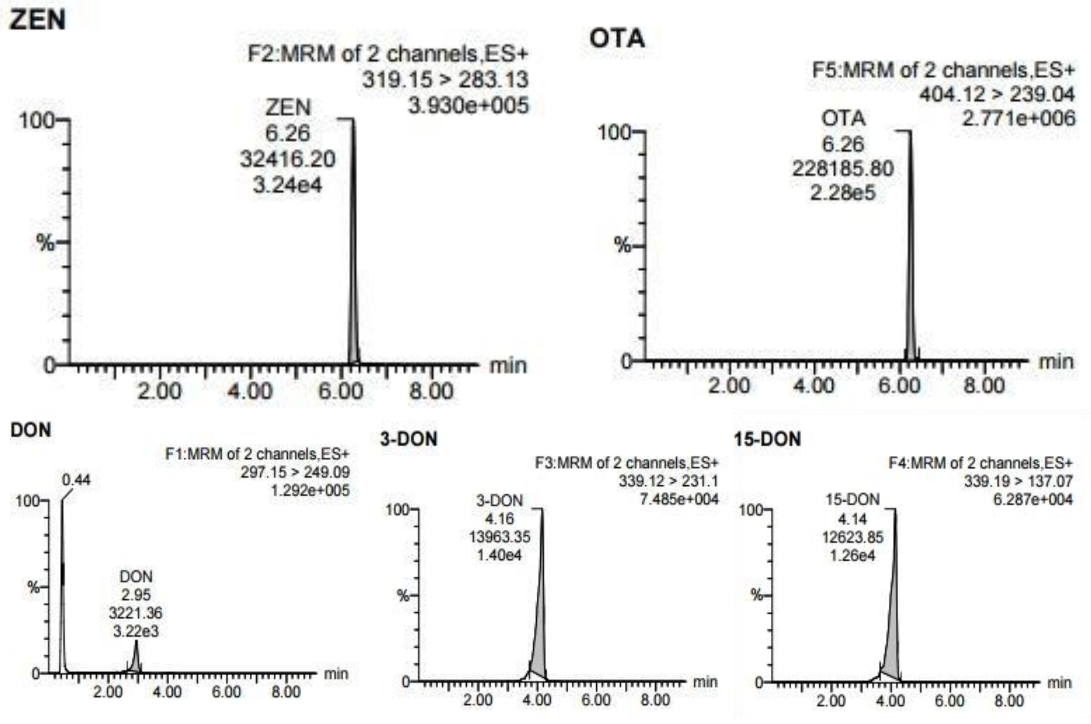

Figure S3. Mass spectrogram about quantitative ion pair of five mycotoxins.
